# A Distinctive Histological Form of Squamous Cell Carcinoma With Perineural Invasion

**DOI:** 10.7759/cureus.25111

**Published:** 2022-05-18

**Authors:** Jason T Bard, Stephan F Duran, Alice A Roberts, Joseph M Wentzell

**Affiliations:** 1 Dermatology, Eastern Virginia Medical School, Norfolk, USA; 2 Dermatopathology, Eastern Virginia Medical School, Norfolk, USA

**Keywords:** fried egg, perineural tumor, squamous cell carcinoma, skin cancer histology, perineural invasion, scc-squamous cell carcinoma, squamous cell carcinoma (scc)

## Abstract

A modest proportion of individuals diagnosed with squamous cell carcinoma (SCC) display perineural invasion (PNI), the neoplastic invasion of one or more nerves. It is associated with a marked increase in mortality in patients with SCC and is oftentimes only diagnosed after a significant invasion occurs. An 84-year-old male, otherwise in good health, presented to us with a fast-growing, 3-cm nodule on his right malar region associated with paresthesia and radiating pain. Biopsy of the lesion revealed moderately differentiated infiltrative squamous cell carcinoma, which was later discovered to involve the perineural fascia of the trigeminal nerve. Excision of the infraorbital nerve and maxillary bone was performed to remove the tumor, with the resulting defect later reconstructed. Here, we present findings of SCC with unique histological features predictive of potential PNI. These features include a rim of cuboidal cells which quickly transition into a well-differentiated, eosinophilic parakeratotic core, reminiscent of a “fried egg” appearance. Awareness of these histological findings may allow clinicians to detect PNI in patients with SCC before widespread and irreversible involvement.

## Introduction

An estimated 20% of all malignant cutaneous neoplasms are diagnosed as squamous cell carcinoma (SCC), totaling to over one million individuals in the U.S. each year [1]. About 5% of these cases display perineural invasion (PNI) annually. The most widely used definition of PNI is in relation to a “tumor in close proximity to a nerve and involving at least 33% of its circumference or tumor cells within any of the three layers of the nerve sheath” [2-4]. This is an important characteristic to be vigilant for, as it is associated with an increased mortality in patients with SCC. In one study, 46% of patients with SCC displaying PNI had passed away or were alive with recurrence at two years’ follow-up, compared to 9% of patients with SCC without PNI. The disease-specific death rate had been reported as 16% for cutaneous SCC with PNI versus 4% for cutaneous SCC without PNI [5]. Currently, clinicians typically diagnose SCC with PNI incidentally, after the fact. Here, we present a unique finding seen in SCC that we believe may represent distinctive histological features with affinity for perineural invasion. This article was previously presented as a meeting poster at the Eastern Virginia Medical School Research Day on October 15, 2021.

## Case presentation

An 84-year-old male with Type II skin presented to a Mohs surgeon for evaluation of a fast-growing nodule on the right malar region during the preceding six to eight weeks. The nodule was poorly demarcated and measured approximately 3 cm in diameter. This was associated with paresthesia and a “shooting” pain from the right malar region to the right upper lip and alveolar ridge. A subtle decrease in right upper lip motor function was also noted. The skin displayed only moderate sun damage. The patient otherwise felt in his normal state of good health and was not in acute distress. The physical exam was unremarkable and indicated no dysphagia, hoarseness, sinus tenderness, cardiac thrills, shortness of breath, lymphadenopathy, nor muscular dysfunction.

A skin biopsy was performed and demonstrated what was diagnosed as moderately differentiated infiltrative squamous cell carcinoma. However, the pattern was not easily classified because the characteristics ranged from poorly differentiated to well-differentiated throughout the specimen. A CT scan that was performed did not reveal any sinus invasion. An initial stage of Mohs surgery was undertaken on the patient’s behalf. Two excised specimens from the first stage of surgery were frozen in a cryostat, sectioned on a microtome and then mounted on slides for hematoxylin and eosin (H&E) staining. They revealed a keratin-producing proliferation of atypical keratinocytes penetrating into the underlying dermis and muscle. Multi-focal, widely spaced discontiguous foci of tumor suggested in-transit metastases. Tumor growth was identified within the perineural and perivascular fascia of the trigeminal nerve and its artery. Expansive intraneural tumor growth, and possible growth within perineural lymphatics, completely obliterated portions of the trigeminal nerve while smaller cutaneous nerves were spared.

The second stage of Mohs surgery extended the excision to the malar periosteum. Two excised specimens from the second stage displayed similar tumor morphology and histopathology to the first-stage specimens. It was evident that the tumor had extended through the infraorbital foramen into the infraorbital canal. An otolaryngology consultation was obtained, and a wide local excision was carried out, including bone resection and further excision of the trigeminal nerve. Surgery included excision of the maxillary bone and infraorbital nerve. The nerve’s gross and microscopic appearance showed tumor involvement. The facial nerve was anatomically identified and preserved. The patient also underwent a right-sided neck dissection, superficial parotidectomy, and reconstruction with a cervicofacial rotation and advancement flap. The bony defect was reconstructed with an area of de-epithelialized skin. Adjuvant proton therapy was then performed over the next seven weeks to prevent reoccurrence of the neoplasm.

**Figure 1:**
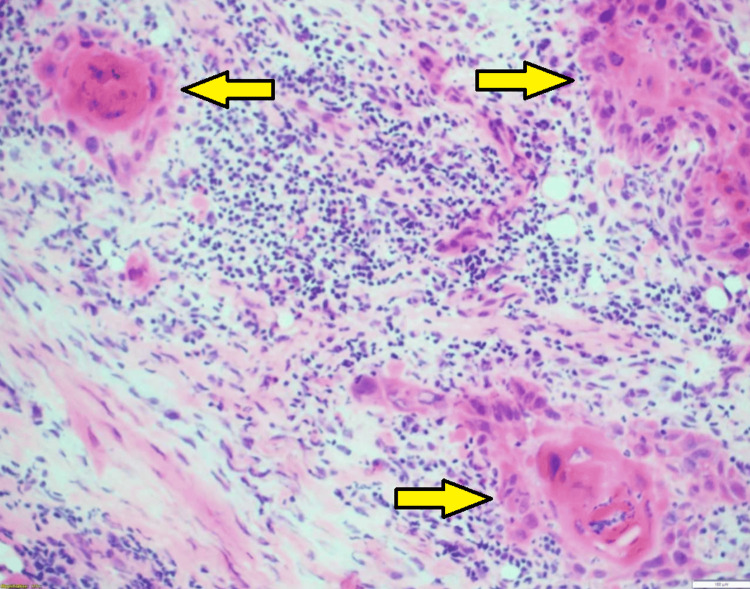
Thin rim of cuboidal cells abruptly transitioning to an eosinophilic, parakeratotic core, resembling "fried eggs" (H&E, 100x).

**Figure 2:**
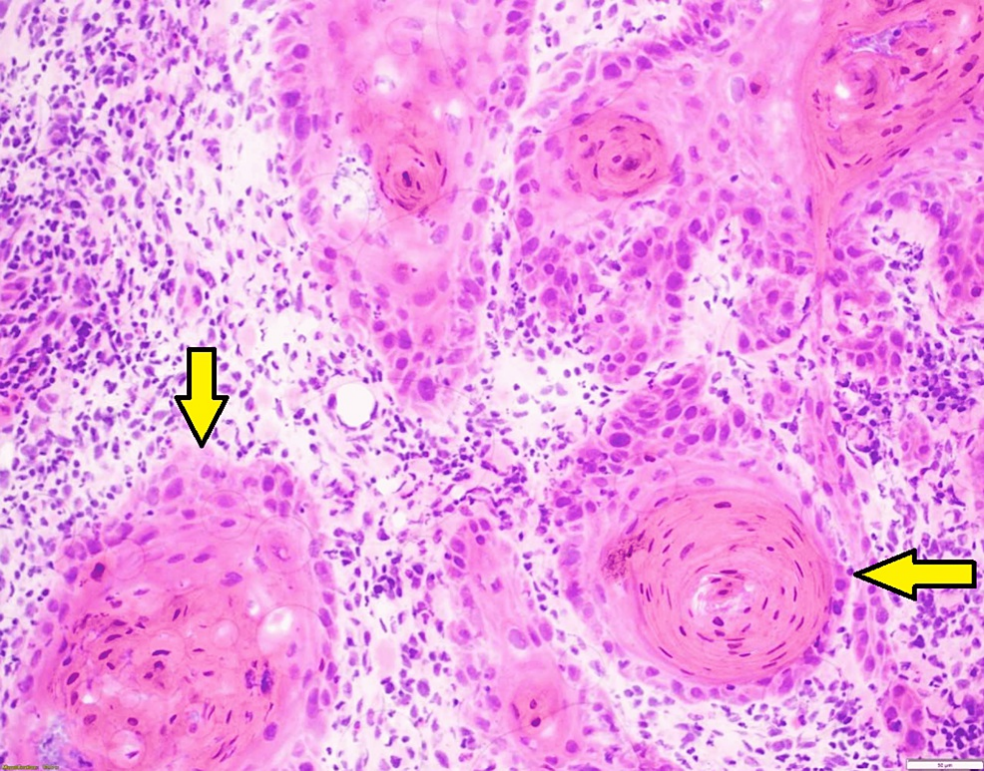
Multiple parakeratotic foci surrounded by eosinophilic cuboidal keratinocytes, resembling "fried eggs" (H&E, 200x).

**Figure 3:**
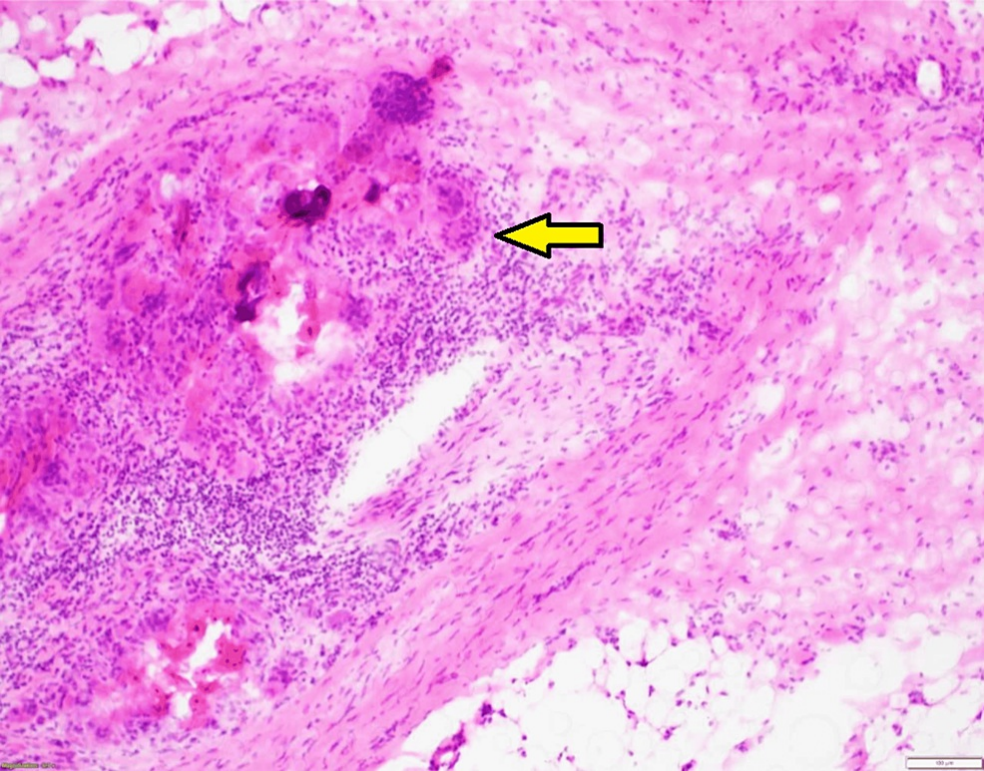
Spindle cells representing a cranial nerve; note the invading keratinocytes (H&E, 40x).

The key findings from biopsy highlight how the association of poorly differentiated cells to well-differentiated cells created a repetitive and recognizable pattern highly predictable of PNI. The recognizable and predictive pattern feature starts with a thin rim of cuboidal cells. Within one or two layers, these cuboidal cells abruptly transition to a well-differentiated, strongly eosinophilic parakeratotic core with only occasional complete orthokeratosis in the most superficial components. The pattern can be reminiscent of the parakeratotic maturation seen in keratoacanthoma. The overall pattern of the tumor proliferations is analogized as having a “fried egg” appearance, as seen in Figure 1. The characteristic pattern of growth is typically found throughout the tumor, from the most superficial components to the most deeply penetrating. The size of the individual growth centers that form the pattern is the most variable feature. Eosinophilic cuboidal keratinocytes surrounding parakeratotic foci are noted in Figure 2, while spindle cells representing a cranial nerve are displayed in Figure 3.

## Discussion

Classification of tumor invasiveness can be determined by microscopic tumor thickness, tissue level, the character of tumor budding, lymphovascular invasion, and perineural invasion (PNI) [6]. These factors are important while considering histopathological differentiation. Peripheral nerves consist of nerve bundles with three layers: epineurium, perineurium, and endoneurium. PNI usually involves the trigeminal nerve and, to a lesser extent, the facial nerve [6-7]. It is often described as in relation to a tumor near a nerve and involving at least 33% of its circumference within any of the three layers of the nerve [2-4]. If the diameter of the affected nerve is less than 0.1 mm and is not close to the primary lesion, then the presence of PNI is not likely to dampen the prognosis. It was reported that if the affected diameter was less than 0.1 mm, there was a 0% disease-specific death rate. When the affected diameter was greater than 0.1 mm, there was a 32% disease-specific death rate [8]. Another study found that recurrence-free survival rates were lower in patients with SCC with PNI versus patients with SCC without PNI [9]. Sometimes PNI is discovered incidentally, which means it can only be detected by histopathological means, rather than by physical examination or radiographic findings. Considering this in conjunction with the poor prognosis seen in SCC with PNI, it is therefore crucial to be aware of any histological patterns associated with this case.

We present findings of an aggressive SCC with potentially unique histology that may represent SCC with affinity for perineural invasion. Histologically, perineural involvement in SCC has most often been described by its differentiation (i.e., typically poorly differentiated squamous cell carcinoma) or its growth features (i.e., typically infiltrative) [5-6]. However, growth pattern, as a repetitive structural analogy, has not been associated with perineural SCC in a useful, predictive way, to the best of our knowledge. In an unpublished study of Mohs surgery for one hundred sequential large perineural SCCs, one of the authors (JW) found the histopathologic patterning, or silhouette, present in this case study to be the most predictive feature for perineural involvement, more so than histopathological differentiation. Anneroth’s multifactorial grading system, deemed as the standard grading system in oral SCC, takes into account the degree of keratinization, nuclear polymorphism, number of mitoses, pattern invasion, stage invasion, and lymphocytic infiltration. Under this system, pattern invasion in highly malignant tumors is described as cellular dissociation in small groups of infiltrating cells [10]. However, we wish to highlight the specific parakeratotic foci surrounded by cuboidal cells, which is not emphasized in the current literature regarding oral SCC, as a predictive quality of PNI.

Because this growth pattern encompasses a range of cell differentiation, past authors who have attributed perineural growth to "poorly differentiated" SCC may have included "fried egg" SCC in their studies, while perhaps not recognizing the predictive quality of this pattern. Although it may be seen in well-differentiated SCC, it can be predictive of potential perineural growth, making recognition of the pattern a potential adjunct to tumor localization and management.

## Conclusions

This case appears to depict distinct histological findings of PNI that have not yet been reported, to the best of our knowledge. It displays SCC with PNI consisting of multiple parakeratotic foci surrounded by eosinophilic cuboidal keratinocytes, resembling a targetoid or “fried egg” pattern. SCC with PNI is typically discovered as an incidental finding, yet this case presents a recognizably associated growth pattern, more specific than what is discussed in the current literature. This observation may allow clinicians to earlier distinguish an affinity for potential perineural involvement in SCC.
